# Topics of Public Interest

**Published:** 1917-09

**Authors:** 


### TOPICS OF PUBLIC INTEREST

**Revision of Rank for the Medical Dept., U. S. Army.** Senator Owen presented an amendment July 20, providing in future for distribution of medical officers in the regular army, as follows: Major Generals and Brigadier Generals, each ¼% of total number of officers which shall be 7: 1000 men in the regular army and national guard; Colonels 4%;

Lt. Colonels 8%; Majors 23.5%; Captains and First Lieutenants, each 32%. The relative grades of the officers of the Medical Reserve Corps shall be the same as the grades of the Regular Army. According to one construction, this last provision seems rather too liberal, certainly if men who have given their entire lives to the regular service should be passed over and the highest ranks awarded to men only temporarily in the medical service. It will be noted that, with allowance for mortality, this amendment virtually assures every competent, surviving member of the Medical Corps, the rank of Major in due time without the long blocking of promotion by rapid diminution in number of higher positions, such as has prevailed in the past. With the Medical Corps on its ante-bellum scale, of something over 600, it would provide 3 generals of the two grades, instead of one of the lower grade. In this connection, it may be stated that the Surgeon General has regularly held the rank of Brigadier, Major General Gorgas owing his higher rank to his especially distinguished services in the sanitation of the Canal Zone. So far as can be judged at present, the total number of medical officers will be 10,000 within a few months, so that there will be 50 Generals, equally divided between the two ranks, instead of one for the entire Corps.

---

**Air-Raid Mortality, London.** During the first three years of the war, 366 death and 1,092 non-fatal casualties occurred. Meantime, 2,412 deaths and 7,863 injuries occurred from ordinary street accidents. Cities of N. Y. State, in 1915, reported an average of 13.2 deaths per 100,000 population, from street car, automobile and vehicular accidents, not to mention other causes included in the more general heading for London. As the London statistics apply to the Metropolitan area, with a population of over 7 million, it can readily be calculated that London, with air raids, is slightly safer than New York.

---

**Centralization of Municipal Medical Work, Buffalo.** The West Farm Hospital, to be opened soon with 1000 beds, the Ernest Wende (contagious) and the Municipal Hospital as well as the 5 city dispensaries have, by an ordinance recently adopted, been placed under a single board of control, with Dr. Walter S. Goodale, Superintendent of City Hospitals and Dispensaries. The ordinance provides for 5 city physicians and it is expected that the present incumbents will be continued. There are 140 other city employees connected with the hospitals and dispensaries. No radical change in the actual working of the units is contemplated.

**The Mental Hygiene War Work Committee** of the National Committee for Mental Hygiene is anxious to obtain the names of psychiatrists and neurologists who are willing to give part-time service in the examination of National Guard troops in their vicinity. The recent decision of the War Department to examine the National Guard troops in their armories before sending them to camp, makes it necessary to secure at once a large number of examining physicians. To meet the situation the Surgeon General of the Army has arranged to accept for this work qualified physicians on contract. A physician may contract for specified duty, at a specified place, for a specified time, or for part-time. This latter provision makes it possible for many physicians who cannot take out commissions, or who cannot give all of their time to the work for a period of months, to give part-time each week. Further information can be received from Dr. Frankwood E. Williams, Vice-Chairman of the Committee, 50 Union Square, New York City.

---

**Bulletin.** Dr. Pearce Bailey of New York, Chairman of the Committee on Furnishing Hospital Units for Nervous and Mental Disorders to the United States Government, a subcommittee of the National Committee for Mental Hygiene, has been invited by the Surgeon General of the United States Army to accept a commission as major and to come to Washington as personal advisor to the Surgeon General in all matters pertaining to phychiatry and neurology. Major Bailey is now on duty in the Surgeon General's office. Dr. Frankwood E. Williams, Associate Medical Director of the National Committee for Mental Hygiene, has been appointed Vice-Chairman of the committee and placed in charge of the work in the New York office.

---

**Increase in Longevity.** Wm. H. Davis, Chief Statistician of the Dept. of the Census has kindly furnished the following statistics:

U. S., practically the entire white population (Meech) 1830, 1840, 1850, 1860, average life of males, 41.01 years; of females, 42.91.

U. S., registration states, white and colored, 1901, males, 47.88; females, 50.70.

U. S., registration states, white and colored, 1910, males, 49.86; females, 53.24.

Massachusetts, entire population, 1890, males, 42.50; females, 44.46.

Massachusetts, entire population, 1901, males, 46.07; females, 49.42.

Massachusetts, entire population, 1910, males, 49.33; females, 53.06.

The close agreement between national and state statistics is corroborative of the real optimistic significance of the figures and the contrast with the statistics 1830-1860 minimizes somewhat the improvement, on account of the inclusion in the more recent statistics of the various non-Caucasian races. It may be inferred that there was no notable gain from 1830 to 1860 so that it may be concluded that the longevity was the best that could be expected without definite scientific control of sanitation and marked therapeutic advances. The improvement subsequently noted is progressive and has occurred in spite of a gradual increase in density of population which, without counterbalancing favorable factors, would certainly have resulted in decreased average longevity. It is significant that the longevity of females exceeds that of males, throughout, in spite of well known physical sexual handicaps. With due allowance for the probable influence of average better behavior of the female sex, we think this must be regarded as a practical result of chivalry. From the standpoint of the medical profession, it is particularly gratifying to note that the average longevity of females has been increased by over ten years between 1860 and 1910 and that the longevity of males has been increased by less than nine years. This difference can not be attributed to infancy and early childhood, when the sexual differences are of practically no importance, nor to general improvement of sanitary and living conditions. It may fairly be claimed that it is due mainly to actual advance in therapeutics, and probably very largely along the lines of obstetrics and gynaecology.

---

**The National Board of Medical Examiners,** Dr. J. S. Rodman, Sec., Philadelphia, reports that 12 candidates were examined in Washington, June 13-21, of whom 9 passed and are therefore, eligible to appointment to the regular Medical Depts. of the Army and Navy, without further professional examination. 12 other candidates were eligible but were prevented from being examined by active military service. The next examination will be held in Chicago, Oct. 10-18, and there will be an examination in N. Y. early in Dec.

---

**Narcotic Drugs.** It is obvious that, especially among the newer synthetics and proprietary compounds known by more or less suggestive names, there are some drugs which relieve pain which are not in any sense habit-forming or dangerous and which therefore do not come within the application of the law while there are others which are mere mixtures of narcotics or which contain as one or the sole ingredient, a slight chemic modification of an officinal narcotic and which, therefore, are subject to the law in all respects. We have to secure from the national and the state authorities, lists of drugs as to which this question might arise, distinguishing between those coming within the scope of the law or beyond its jurisdiction. Unfortunately, neither source of legal administration is a source of information and we advise the utmost caution and, especially, an inquiry into the actual composition of a pain relieving drug, before prescribing. As the application of the law depends upon rulings which have not been in all cases made by those familiar with pharmacology, various inconsistencies and contradictions have occurred and we would warn that a proprietary, advertised in good faith as not amenable to the narcotic laws, may by a subsequent ruling become so.

---

**The State Civil Service Commission, Albany,** announce various examinations in which medical men would be interested but the information is received too late for publication before the date of the examinations. We would advise our readers who are especially interested to apply directly for information blanks.

---

**Occupation in Relation to Tuberculosis.** The Bureau of the Census announces that, it expects to issue a bulletin based on the statistics of 1918 and it particularly urges physicians to note the occupation on all death certificates (and, by implication, on morbidity reports, etc.). It occurs to us that it would be well to establish a proper designation for many women and girls not engaged in "gainful" occupations and only by an extension of the term, to be designated as housewives; also that some plan should be formulated to indicate the essential hygienic conditions of work, for instance, to show whether a clerk is engaged in indoor or outdoor work, and to show how superintendents, managers, secretaries, vice-presidents, etc., are actually employed.

---

**X-ray Examination of Soldiers.** 1030 members of the 69th Reg. N. G. N. Y. have been submitted to examination. Of the first 600 plates examined, 22 showed active tuberculous lesions sufficient to disqualify and 18 old lesions.

---

**Fecundity of Twins.** Some months ago, we published an abstract of an article by J. P. Foster, claiming that of twin sisters, one is always sterile. The Medical World published the same claim and in the August issue, prints 8 letters, citing 15 pairs of fecund twin sisters. We wish we could secure the same spirit of co-operation.

---

**Commissions in Medical Reserve.** The following, in addition to those previously noted, have been announced: Marshall Clinton (in charge of Base Unit No. 23), Major; John F. Fairbairn, Major; Nelson G. Russell, Milton M. Goldberg, Wm. Ostrow, Walter L. Hachemer, Albert A. Gartner, G. J. Geisler, Richard N. De Niord, 1st lts., all of Buffalo.

---

**The Buffalo Assn. for the Relief and Control of Tuberculosis** has occupied the building 175 E. Swan St., where the tuberculosis dispensary is also conducted. About 135 cases are examined each month. Those intended for the J. N. Adam Memorial Hospital are submitted to further examination by the Superintendent, Dr. Horace Lo Grasso.

---

**Tetanus Bacilli in Court Plaster.** In regard to the press report that peddlers were selling court plaster infected with disease germs by German sympathizers, tests made of suspected samples at various places have shown the presence of tetanus bacilli. This does not by any means establish the contention of a deliberate plan to disseminate disease as such germs might easily be introduced in dust, especially in samples peddled from door to door in the country. Aside from a proper reluctance to admit so nefarious a practice even by an enemy, it is highly improbable that tetanus would have been selected, as this is a disease whose germs are widely distributed everywhere where horses exist and manure has been employed for the soil. Moreover, the chances of infection of superficial wounds, even when sealed by court plaster, would be comparatively small. Granted the intent to go contrary to the established precedents of civilized warfare, and it must be confessed that many facts establish such an intent, it is altogether probable that our enemy would have selected a far more efficient means and would not bring coals to Newcastle by impregnating plasters with tetanus bacilli. The moral against buying any kind of domestic medical supplies from irresponsible dealers should, however, be emphasized.

---

**Prisoners Held by Central Powers.** Figures in parenthesis by Germany. Russians 2,080,699 (1,212,007), French 368,607 (367,124,) Serbian 154,630 (25,879), Italian 98,017 (none), Roumanian 79,033 (10,157), British 45,241 (33,129), Belgian

42,437 (42,435), Montenigrin 5,607 (1). From the standpoint of the holders, the prisoners are divided as follows: Germany 1,690,031, (17,474 officers), Austria 1,092,055, Bulgaria 67,-582, Turkey 23,903, total 2,874,271 including 27,620 officers.

---

**Medical Reserve Officers.** The American Medical Editors' Assn. has pledged its support to the Surgeon General. The country still needs officers for the Medical Reserve Corps. It should be a matter of pride to every physician that the whole system of an Officers' Reserve Corps originated with the Medical Dept. of the Army. Do not delay volunteering because of delays in assigning other medical volunteers to active duty. In the first place, part of this delay is such as would occur in any business whose office force encountered the sudden demand of an increase from 600 to 20,000 and whose total employees were similarly to be increased from about 1000 to possibly 150,000. In the second place, about half of the 11,000 volunteers for medical reserve duty failed to make good when called—and the word called may be construed in a figurative sense. In the third place, the age group 21-31 not only fails to represent the active medical profession as it does the manhood of the country generally but does not include as many physicians as are needed. Any physician up to 55 is acceptable, if otherwise in good condition.

Medical Reserve Officers are commissioned in three grades: First Lieutenant at $2000, Captain at $2400, Major at $3000. Only the older and especially experienced may expect Captains' commissions and only those who are very distinguished, Majors'. Perquisites, excess pay of 10% for oversea service and lack of opportunity to spend money, render these salaries more liberal than they seem. While in service, these officers enjoy all the advantages of regular army officers, including disability pensions and some form of insurance will probably be provided by the government. We do not believe that many physicians are cowardly but it is allowable to reiterate that the statements as to risk have been greatly exaggerated. The medical profession of Great Britain has not been wiped out; the surgeons in the British forces have not equaled the total number of medical men in the whole Empire and 12,000 of them have not been killed or even wounded. Just 195 allied surgeons were killed on the western front up to June 27 (three years less about five weeks). In passing, it may be complained that these and similar alarming rumors have been due to the policy of converting news into propaganda, of scaring when there seemed to be a little apathy and of reassuring when a state of too great alarm was developed.

Application blanks for the Medical Reserve Corps may be obtained of the Surgeon General, Washington, of Capt. Herbert M. Smith, M. D., Buffalo, or of this office.

We regret that statements have been made that the medical officer can live on $25 a month and send $150-$225 a month to his family. Barring exceptional conditions, it is neither true chivalry nor good economics. Both sexes and all ages must sacrifice something for patriotism. The fact is that there is considerable profiteering by private firms catering to officers, that the enormous demand for cloth and other material has resulted in an inevitable increase of prices generally and that there is a shortage of many things normally carried in stock in the Quartermaster's Dept. On the other hand, the war regulations proscribe civilian dress, dress uniforms, side arms and, with good sense and consideration, the equipment actually required for Medical Reserve Officers has been reduced to a minimum, of which a list is furnished when commissions are sent. The estimate of $350 for an officer's equipment is unnecessarily high under these circumstances. Part of the equipment can be obtained of local quartermasters and most of the remainder of the Depot Quartermaster, 2600 Gray's Ferry Road, Philadelphia. Uniforms should, of course, be re-fitted by one's own tailor or the cloth may be obtained at the above address. Mess expenses in training camps are $0.75-$1.00 a day. All of the personal equipment, up to cots, bedding, medical supplies, etc., can be carried in an ordinary steamer trunk and suit case or large satchel, and we understand that the bedding and tent equipment may be secured at camp or at stations to which officers are ordered. A fair estimate of necessary preliminary expenses for the minimum equipment is $150. About as much more would be needed to cover current and traveling expenses till the first pay is received. Thereafter, something like $50 a month should be held for current expenses. We understand that, while food and other prices have advanced in Europe, they are not yet so high on the average as in this country. Owing to the maintenance of a fairly stable battle line and of the gold standard of money, the extravagant prices familiar from the accounts of Civil War officers, will probably not be encountered unless very occasionally.

One thing should be very clearly understood by candidates for the Medical Reserve. A very different form of patriotism is required from that exemplified by some civilians who ride around in autos decorated with claims of safe and glorious services, advice to others to enlist or conspicuous notices that they have invested an unspecified amount in Liberty bonds. Don't volunteer unless you mean to accept orders. The Surgeon General does not intend to waste good medical material by unnecessary risks nor good fighting material by ordering internists to perform capital operations. He needs different kinds of skill for different duties—even obstetrics, gynaecology and paediatrics are included in the duties of the Medical Dept. But fine discriminations of specialism cannot always be made, the prudent man who would prefer safety far from the firing line may be ordered to the front of the front and the man who prefers a career of moving picture heroism, may be ordered to fill the place of a more experienced officer and to do very humble routine work in a post in this country.

---

**The National Committee for Mental Hygiene** has issued a circular in anticipation of duties in regard to detecting nervous diseases in the draft, and the prophylaxis and treatment of the various neuroses and lesions that may be expected in military service. The circular is too lengthy for publication but those interested should apply for a copy to the Associate Medical Director, Dr. Frankwood (sic) E. Williams, 50 Union Square, N. Y. City.

---

**Diminished Casualties for France.** The French Army now amounts to a little less than three million on the western front. Casualties (killed, missing and prisoners, apparently not including wounded unless dying) amounted to 5.41% of the total mobilized strength for the battles of Charleroi and the Marne; 2.39% for the first half of 1915; 1.68 for the second half; 1.47 for the first half of 1916; 1.28 for the second half. The last annual rate is 2.75%. It is impossible to state what proportion consists of deaths and what of capture. A fair estimate is that 1.5-2% means military deaths, from 3 to 4 times the average mortality in peace for men of the 20-30 age group.

---

**The National Surgical Dressings Committee** has become a component organization of the Red Cross, this being a step in the direction of unifying all of the various war relief organizations, in the interests of economy and efficiency.

---

**Draft of Physicians Probably Unnecessary.** At the risk of contradicting statements made elsewhere, we would state that the latest obtainable information shows that, of a total estimate of 20,000-25,000 surgeons ultimately needed by the U. S. (this estimate obviously applying to the completion of an army of 2,000,000 or more) 16,000 applications for the

Medical Reserve Corps have been received, not to mention the fact that the National Guard amounting to about 500,000 is supplied and that the Regular Army of about 300,000 is at least half supplied by permanently appointed surgeons. Nearly 9,000 commissions have been accepted from among 14,000 recommended as fit, out of the 16,000. About 2,000 commissions have either not been issued or have been issued too recently to expect a response except from those of unusual promptness. By the time this is in print, the probability is that at least 12,000 of the 20,000-24,000 medical officers ultimately needed, will be available for duty. Meantime, applications are coming in at the rate of 100-150 a day. It should be understood that a good many physicians, who are inclined to enlist, must make careful preparation in various ways before they can even volunteer. Many others are deterred from immediate action—though they should understand the advantage to the Government as to the individual of being able to estimate its resources in advance of immediate needs—because they note that their confreres have waited weeks for commissions and other weeks for orders. There is every reason to believe that an adequate number will be secured by volunteer methods, in amply time. However, those who are delaying, should take the initial step promptly, realizing that the work of enrolling and assigning to active duty is necessarily tedious.

---

**Proposed Increase in French Food Ration.** The demand has been made for an increase from 300 grams to 500 grams a day. This is a dietetic puzzle. Excepting clear fats and oils which, of course, can be used only to a limited extent, very few even of the most nutritious food stuffs yield more than 3.5 calories per gram and the minimum average ration, in calories, is about 2200. Properly assorted, it would take just about 500 grams a day of chemically pure proteid, carbohydrate and fat—of which only the last two are practically available in pure form for food—to make a day's ration. Taking food stuffs as they run, even eliminating the coarse vegetables, about 1000 grams are required daily.

---

**Government Control of Wheat.** When, a few years ago, we suggested that a return to the old system of public granaries might solve the problem of the cost of living, we meant it in quite an academic sense and were rather surprised to find by calculating that, in regard to finance, provision of containers and preservation against decay, the proposition was feasible. Under the impetus of war, steps have actually been taken for the government to buy and sell at reasonable prices, the entire wheat crop. If nothing further is done, such action will automatically control to a considerable degree and quite directly, the prices of all other cereals. As a precedent, it will very likely lead to similar control of other staples. If so, it may be confidently asserted that the war will pay for itself, so far as the people are concerned, no matter what it costs. This statement must be taken in a purely economic sense.

---

**Speculative Food Waste.** The deliberate waste or destruction of foods in order to keep up prices, is one of the meanest problems of economics. We suggest the following scientific basis for suitable measures of control. The average day's ration is 2500 calories; withholding nutriment for 40 days, practically always causes death. 100,000 calories, therefore represents a human life. Tables, accurate enough for practical purposes and bearing the endorsement of the government exist, from which the caloric value of a given weight of any food stuff can be calculated. When anyone deliberately, with premeditation, destroys 100,000 calories of food stuffs, treat him exactly as if he had directly instead of indirectly destroyed a human life, with due regard to precedents of peace and war. Differences in degree often virtually constitute differences in kind. Throwing out a crust of bread may be responsible but does not require serious consideration, any more than a kick in the shins or a slap on the face. The mathematic basis suggested affords a practical way of discriminating between mere carelessness in conservation of food and deliberate criminal attempts to put it above the reach of the population.

---

**Mobilizing Reserve Drug Stocks.** So much has been published regarding the scarcity of drugs, their high price, and the absolute lack of certain ones, that we have contemplated the establishment of a clearing house so that physicians and druggists could dispose of drugs for which they have no use and which might be of great value to others. Obviously, in carrying out such a plan, physicians would mainly provide drugs, including proprietaries, foods and mineral waters for sale while druggists would mainly act as purchasers though the latter would have an opportunity to get rid of articles especially ordered for some physician to prescribe and in which he speedily lost interest. Personal inquiry has led to the belief that, except for a few articles to which such a system would scarcely be applicable, we have not yet reached the stage of depletion at which it is necessary "to scrape the barrel." The advance in prices seems to be due mainly to manipulation, with the easy excuse of disturbance of supply and demand and the druggists whom we have consulted feel that it is easier to lose an occasional prescription than to make any organized effort to secure drugs that are not available through the ordinary channels. However, the Journal stands ready at any time to assist in this direction and will receive lists of drugs and analogous articles wanted and for sale, from either physicians or druggists. Under present conditions, we are inclined to believe that the clearing house had better be limited to offers to hospitals, the Red Cross or other philanthropic institutions.

---

**Statistics of Medical Education, 1917.** Total students 13,-764 (11,826 in 1880; 28,142 maximum in 1904, averaging about 25,000 1900-1910). All non-sectarian but 580 homoeopathic and 250 eclectic. (From 1880 to 1907, over 1,000 homoeopathic, 545 to 1,014 eclectic, about 300 of other schools which have disappeared since 1911). Total graduates 3,379 (lowest number since 1880: 3,241). Over 4,000, 1890-1912; over 5,000, 1900-1906, maximum 5,747, 1904. All non-sectarian but 180 homoeopathic and 65 eclectic, formerly sectarian graduates amounted to about 500. 1,099 (32.5%) of this year's graduates had bachelor's degrees, the maximum proportion. In 1910 15.3% of graduates had bachelor's degrees; in 1915, 24.3%; in 1916, 26.9%. The proportion for this year's graduates was 34.4% for non-sectarian, 10.5% for homoeopathic, 3.1% for eclectic. 153 women graduates, 610 students, 1917. From 1900-1907, medical colleges numbered within 2 of 160, (125-127 non-sectarian, 18-22 homoeopathic, 8-10 eclectic, 3-5 physio-medical, etc.) In 1917 (as in 1915) the total was 96, including 9 homoeopathic and 4 eclectic. Tables compiled for 3,015 graduates from 79 colleges give the average age of graduation as 26.4, the same as for 1,668 students of 41 colleges requiring high school education in 1913; 446 students at 14 colleges requiring one year of college work in 1913, gave an average of 1/10 year less; 901 students at 24 colleges requiring two or more years of college work in 1913, gave an average 2/10 year higher. Somehow the argument of undue delay of entrance on life work, does not hold good in practice. In 1917, 2,577 were graduated from Class A colleges, 648 from B and 154 from C. (Acknowledgment to Jour. A. M. A.)

---

**Military Strength of U. S.** Last year, the Regular Army numbered a few less than 103,000 and the effective strength of the National Guard was estimated at about 125,000 though its normal strength was considerably greater. At present, the

Regular Army and the National Guard each number very close to 300,000, there are about 55,000 reserve enlisted men and about the same number of officers in the two armies and in reserve, the aggregate being 710,000. The sea forces number in all, 233,000. The recent increase in regimental strength is being met by enlistments. It is altogether probable that before reaching the drafted National Army, we shall have an efficient land force of at least 800,000 and perhaps an even million.

---

**Reorganization of Army Units.** Rather unexpected radical changes have recently been made and definite information has not been received of the final make-up. In an approximate sense, the following seems to be correct: Each unit, on the basis of the previous wars of the U. S. and the average peace strength, will be enlarged to the size of the next regular unit, the command and responsibilities of an officer of any given rank being correspondingly increased, resulting in a decided economy, both as regards salaries of officers but proportionate number of officers and enlisted men. For example, the old, temporary squad or company division will be more or less definitely assigned to a second or first lieutenant, instead of having the lieutenants as assistants without definite command, and will number 60 or more, which was the old practical company strength. There will be two captains to a company, one apparently having about the same duties as a vice-president, to wait till his senior is killed or incapacitated, and the company equals the former actual strength of a battalion. The major, in charge of a battalion, will command what was formerly a full regiment. Indeed, as the present war is being fought so as to render feasible a scheme for reserve companies to keep the numbers full, the company will be nearly equal to the actual fighting strength of Civil War regiments during a good deal of the time of hostilities. The regiment is to be increased to 3600 men, will include headquarters and machine gun companies and, we understand, artillery and other branches so that it will not only be fully equal in numbers to the old brigade but will be a polycratic unit and will thus adopt to some degree, the conception of the former division as a complete fighting unit. The brigade will be relatively small, consisting of two regiments and as the division is to be limited to 19,000, it looks almost as if the brigade itself would be found to be an unnecessary and cumbersome intermediate unit. The corps will be restored and will be of about the size of the Civil War army in any single terrain or of the ''field army'' which never really passed the paper stage. The economy of managing what was formerly a rather large brigade, including three regimental commands, with a single regimental staff will be considerable. The question may arise whether it is quite fair to the officer to promote him in regard to the number of men whom he is to command without an increase of rank or pay or whether, as a matter of efficiency, the extension of executive authority will be feasible. On the other hand, the present war involves a fixity of terrain and of routine not previously experienced while the authority and efficiency of non-commissioned officers has been gradually increased.

---

**Proposed Exemption of Medical Students From Draft.** A movement has been made to secure legislation. There are (A. M. A. statistics) 10,385 students remaining from last session's enrollment, of whom about 75% are liable to the draft though, of course, only about 20% would be drafted on the basis of a National Army of two million. There are obvious reasons why the exemption, if passed, should not apply to future matriculants. There would be the temptation to slackers and the future medical profession would be injured both quantitatively and qualitatively. The argument that, with an annual graduation of slightly over 3,000 physicians, there is already a demand for 4,000 internes annually, does not appeal to us. Whether young men of an average age of 26 and a quarter to a third of college education beside their technical training, should be expected to work a year for experience plus board and washing and, perhaps an honorarium of $2 a week, is a question that we think should be answered as it is for teachers, engineers, ministers, plumbers and carpenters. The economics of the present interne system is the natural result of a disproportionate supply and merely one example of the condition of the medical profession. Without any seeking on our part, the War affords us a golden opportunity to hasten the righting of an economic wrong that would not be tolerated in any other profession or trade.

---

**Poliomyelitis.** One mild case has been reported for Buffalo, up to Aug. 22. Very few cases have been reported for the state or the country generally, as compared with the epidemic of last year. Why? Did the quarantine and sanitary measures that apparently had no effect last year, except in producing a minimum general death rate among young children in spite of the epidemic of this disease, stamp out fomites? Was the susceptible material exhausted and, if so, what constitutes the aggregate small proportion of susceptible individuals? Was there some insect carrier prevalent last year that is absent this year? Are the climatic differences between the two years the reason, either as acting on an insect host or in other ways? Is the disease not specific in the clinical sense and is it still present in a form not recognized?

---

**Suppression of News.** Several items, including personal notes of appointments to the Reserve Corps, etc., have been omitted. While we do not flatter ourselves that the publication of such matter in a monthly journal would have much military importance, we wish to conform to the spirit of the request of the government. In some instances, also, private reasons exist for not divulging military service. We have, therefore, adopted the general policy of refraining from publishing items which have not already been given publicity or which are not personally sent for our columns.

---

**Limitations to Use of Army Titles by Red Cross.** Henry P. Davison, Chairman of the War Council of the American Red Cross, announces that military titles are conferred solely as a matter of protection and are to be used only during actual service in the war territory, not in this country. (In plain words, we understand that the military titles are given simply to remove the excuse for treating Red Cross workers as snipers).

---

**Small Pox.** One case has developed in Buffalo, in a negro, one of a construction gang from Erie, Pa. Chicago has had quite an epidemic from a similar source.

---

**The Erie County Morgue** is to be improved.

---

**German Losses.** English tabulations of lists issued by the German government up to July 1 (not necessarily including recent losses nor representing the actual total) show: Killed (including fatal wounds) 1,032,800; Died of Sickness 72,960; Prisoners and Missing 591,966. (Note that the Allies' statistics published in another paragraph show 1,690,031 German prisoners) ; Wounded 2,825,581. Taking the German reports of deaths and, obviously, there can be no counting the enemy's dead in this war, the Allies' reports of prisoners and estimating 10% of wounds as producing permanent incapacity, the definitive losses of Germany amount to considerably over 3,000,000, out of an initial "militia of about 7,000,000. The annual increment of males maturing to military age is about 1% of the population (about 67,000,000), about 500,000 annually being available for military service. The net loss for the slightly less than three years has, therefore, been about 1,500,000.

---

**Niagara County Tuberculosis Hospital.** Bids aggregating $108,960, have been received for the 95-bed hospital planned for the old poor-house farm near Lockport. The appropriation was $100,000 but the estimate exceeded the bids.

---

**Draft Statistics.** Of 100 summoned to Olean, 81 reported and 64 were passed.

---

**The Medical Division, U. S. Reserve Corps,** numbered as follows, Aug. 17: Majors, 350; Captains, 1,367; First Lieutenants, 7,158.

---

**Base Hospital No. 23,** was mobilized at Ft. Porter and began active training, Aug. 23. The medical staff is as follows: Major Marshall Clinton, commanding officer; Captain Lorenzo Burrows, Jr., adjutant; Captain J. H. Hickey, quartermaster; Major Nelson G. Russell, Major John F. Fairbairn. Captains John B. Betts, H. A. Smith, D. C. McKenny, F. P. Goodwin, F. W. McGuire, J. P. Brennan, R. E. DeCeu, Baldwin Mann. Lieutenants H. F. May, W. L. Machemer, H. C. McDowell, J. A. P. Millet, A. A. Gartner, R. N. DeNiord, A. W. Thompson, H. K. Hardy, T. F. Donovan, H. C. Fairbanks. Dental Surgeons, Lieutenants C. L. Storms and E. J. Knoche. The trained nurse staff numbers 65, as follows: Mary Mahl, Mabel Caldwell, Matilda Sturtzer, Mara Fellers, Frances Welker, Elizabeth Welch, Clara Judson, Josephine Stevens, Anna McDonald, Jennie McNaughton, Margaret Bailey, Ella Renn, Sarah Smith, Elsie Sarge, Jessie Urquhart, Edna Reese, Emma Snyder, Edith Zamstone, Edith Switzer, Jessie Murray, Mary Meek, Josephine Briody, Mildred Beamer, Jean Crawford, Mina Ross, Margaret Bruce, Gladys Baker, Helena Meadows, Frances Evans, Anna Austin. E. Blanche Augustine, M. Volland, Mildred Davis, Lilly Strange, Mabel Waltham. Mary Winter, Grace Williams, Sarah Campbell, Mary L. McIntosh, Bertha Bezitt, Mabel Gardner, Mildred Englend, Martha Morningstar, Cora McDade, Alice Wallace, Edith Fenwick. Margaret Hennessy, Josephine Ballou, Ada Hamilton, Nora Kraft, Clara Dudley, Josephine Hull, Maude Bell, Mabel Barr, Luella Clark, Carol Schmid, Norrine Walsh, Florence Carpenter, Dora Scherur, Anna C. Minet, Evelyn C. Carney, Edna Crawford, Kathleen Munschaw, Nellie Donovan. There are 150 enlisted men and a clerical staff.

